# Protocol for a pilot randomized controlled feasibility study of brief interpersonal psychotherapy for addressing social-emotional needs and preventing excess gestational weight gain in adolescents

**DOI:** 10.1186/s40814-020-00578-1

**Published:** 2020-03-20

**Authors:** Lauren B. Shomaker, Lauren D. Gulley, Emma L. M. Clark, Allison M. Hilkin, Bernadette Pivarunas, Marian Tanofsky-Kraff, Kristen J. Nadeau, Linda A. Barbour, Stephen M. Scott, Jeanelle L. Sheeder

**Affiliations:** 1 Department of Human Development and Family Studies, Colorado State University, 1570 Campus Delivery, Fort Collins, CO 80523-1570 USA; 2 Department of Community and Behavioral Health, Colorado School of Public Health, Aurora, CO USA; 3 Department of Pediatrics, University of Colorado School of Medicine and Children’s Hospital Colorado, Aurora, CO USA; 4 Department of Medical and Clinical Psychology and Department of Medicine, Uniformed Services University of the Health Sciences, Department of Defense, Bethesda, MD USA; 5 Department of Medicine, University of Colorado School of Medicine, Aurora, CO USA; 6 Department of Obstetrics and Gynecology, University of Colorado School of Medicine, Aurora, CO USA

**Keywords:** Interpersonal psychotherapy, Depression, Obesity, Pregnancy, Adolescence

## Abstract

**Background:**

Excess gestational weight gain (GWG) in pregnant adolescents is a major public health concern. Excess GWG increases risk of pregnancy complications as well as postpartum and offspring obesity and cardiometabolic disease. Prevention interventions for pregnant adults that target lifestyle modification (i.e., healthy eating/physical activity) show insufficient effectiveness. Pregnant adolescents have distinct social-emotional needs, which may contribute to excess GWG. From an interpersonal theoretical framework, conflict and low social support increase negative emotions, which in turn promote excess GWG through mechanisms such as overeating and physical inactivity.

**Methods:**

The current manuscript describes the design of a pilot randomized controlled feasibility trial of adolescent interpersonal psychotherapy (IPT) to address social-emotional needs and prevent excess GWG. Up to 50 pregnant, healthy adolescents 13-19y, 12-18 weeks gestation are recruited from an interdisciplinary adolescent maternity hospital clinic and randomized to IPT + usual care or usual care alone. IPT involves 6 individual 60-minute sessions delivered by a trained behavioral health clinician during 12-30 weeks gestation. Sessions include relationship psychoeducation, emotion identification and expression, and teaching/role-playing communication skills. Between sessions, adolescents are instructed to complete a daily journal and to have conversations to work on relationship goals. Outcomes are assessed at baseline, mid-program, post-program, and 3-months postpartum. Primary outcomes are feasibility and acceptability based upon rate of recruitment, session attendance, program acceptability ratings, and follow-up retention. Secondary outcomes are perinatal social functioning, stress, depression, and eating behaviors assessed with validated surveys and interviews; perinatal physical activity and sleep measured via accelerometer; GWG from measured weights; and at 3-months postpartum only, maternal adiposity by dual energy x-ray absorptiometry, maternal insulin sensitivity derived from 2-hour oral glucose tolerance testing, and infant adiposity by air displacement plethysmography.

**Discussion:**

This pilot trial will address a key gap in extant understanding of excess GWG prevention for a high-risk population of adolescents. If feasible and acceptable, brief psychotherapy to address social-emotional needs should be tested for its effectiveness to address excess GWG and postpartum maternal/infant health. If effective, such an approach has potential to interrupt an adverse, intergenerational cycle of social-emotional distress, obesity, and cardiometabolic disease among young mothers and their offspring.

**Trial registration:**

ClinicalTrials.gov NCT03086161, retrospectively registered

## Background

An estimated 194,400 babies were born in 2017 to mothers ages 15–19 years in the USA [1]. Major racial/ethnic and socioeconomic health disparities persist in adolescent pregnancy and childbearing. Non-Hispanic Black, Hispanic, and American Indian/Alaska Native adolescents are far more likely to bear children (27–33 per 1000) than Non-Hispanic White (13 per 1000) and Asian adolescents (3 per 1000) [1]. Similar to adults, the majority of pregnant adolescents, approximately two-thirds, gain more weight in pregnancy than recommended by the Institute of Medicine guidelines [2]. This excess weight gain pattern is observed both in adolescents who are lean and those who have overweight/obesity. Moreover, high rates of excess gestational weight gain (GWG) remain consistent when based upon adult-cutoffs for pre-pregnancy body mass index (BMI; kg/m^2^) or on age- and sex-adjusted pre-pregnancy BMI (*z*-score) [2]. Experts have called for recognition of excess GWG in adolescent pregnancy as a major public health concern because it not only increases antepartum and peripartum complications but also increases the risk of postpartum weight retention and future metabolic risk for both the mother and her offspring [3].

### Problem of excess GWG in adolescent pregnancy

Excess GWG in adolescent pregnancy has multiple negative consequences. Excess GWG amplifies the risk of pregnancy complications from pre-existing obesity, including gestational diabetes, hypertension, preeclampsia, and need for Cesarean delivery [4–8]. In addition, excess GWG amplifies mothers’ risk of obesity and cardiometabolic disease after pregnancy [9–12]. The more excess weight gained in pregnancy, the more weight retained in the postpartum, increasing mothers’ long-term risk of obesity and cardiometabolic disease progression [9–12]. In a large investigation of maternal and fetal growth during adolescent pregnancy, adolescents with excess GWG had greater postpartum weight retention, BMI, central adiposity, and peripheral adiposity over a 2-year period compared to adolescents who gained recommended amounts of weight [13]. Pregnant adolescents with excess GWG were nearly 5 times more likely to have obesity approximately 12 years post-delivery than those who gained appropriate weight [9]. While similar adverse effects have been documented in adults [14], excess GWG in adolescent pregnancy may be particularly deleterious [13]. In a study comparing adolescent and adult pregnancies, adolescents with excess GWG continued to gain more weight between an initial and subsequent pregnancy as compared to adults [15]. Adolescence itself is marked by increases in weight and adiposity in females; therefore, pubertal gains in central adiposity are likely exacerbated when pregnancy is timed in adolescence [13].

Beyond the adverse effects on mothers, excess GWG also affects offsprings’ risk of obesity and cardiometabolic disease [7, 8, 16–21]. This intergenerational effect perpetuates a cycle of obesity and cardiometabolic disease in a population at high-risk for social stressors, emotional difficulties, obesity, and preventable chronic illness [22–25]. Offspring of adolescent and adult mothers with excess GWG have higher odds of macrosomia, greater neonatal adiposity, higher BMI in childhood and adolescence, and greater cardiovascular disease risk factors in childhood [7, 8, 16–21, 26]. The “fetal overnutrition” explanation, supported by animal and human studies, is that intrauterine caloric excess creates a metabolic milieu that heightens obesity and cardiometabolic risk, independent of other genetic and postnatal environmental risk factors, through a host of mechanisms such as insulin resistance and subsequent hyperinsulinemia, hyperglycemia, hypertriglyceridemia, increased fat deposition in subcutaneous and intrahepatic depots, changes in appetite regulation, mitochondrial function, impaired functioning of the adipoinsular axis, and epigenetic modifications [27]. Thus, feasible and effective prevention of excess GWG in pregnant adolescents is a priority for reducing adolescent mothers’ obesity and cardiometabolic disease risk, and for disrupting the transgenerational cycle of obesity and cardiometabolic disease affecting offspring of successive generations [28].

### Prevention of excess GWG

There have been few interventions designed to address excess GWG in pregnant adolescents. In adult pregnant women, the standard approach is lifestyle modification intended to promote healthy eating and physical activity. In an expert review of over 50 randomized controlled trial studies in pregnant adult women, existing programs unfortunately were concluded to be costly, time intensive, and limited in effectiveness and scope [29]. In non-pregnant adolescents who have overweight/obesity, lifestyle-based interventions to address weight management face challenges for adherence and effectiveness, particularly in youth from historically disadvantaged racial/ethnic groups [30–34]. Thus, alternative strategies to lifestyle-based approaches that are feasible and acceptable to pregnant adolescents are highly warranted.

**Fig. 1:**
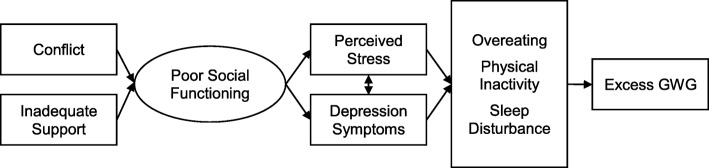
Theoretical model of interpersonal problems and excess gestational weight gain (GWG)

One potentially promising framework is to target the underlying social-emotional contributors to excess GWG in pregnant adolescents. Interpersonal conflict, insufficient social support, and emotional concerns such as depression are highly prevalent in pregnant adolescents [35]. During pregnancy, interpersonal stress increases within romantic partnerships, and potential lack of involvement from the father-of-the-child is related to higher levels of depression symptoms in adolescents [36, 37]. Inadequate social support, perceived stress, and depression symptoms have been associated with excess GWG and postpartum weight retention [38–41]. In pregnant adolescents, prevalence of elevated depression symptoms (14–48%) is doubled compared to both non-pregnant adolescents and to adult pregnant women [42–45]. Adolescents who gain weight rapidly and who have greater total GWG report the highest levels of depression during pregnancy, as compared to adolescents who have slow or adequate GWG [46]. As displayed in Fig. 1, we propose that poor social functioning may contribute to perceived stress and depression symptoms, which promote excess GWG through mechanisms such as overeating in an attempt to cope with negative emotions, physical inactivity, and sleep disturbance [47–52].

Therefore, interventions designed for pregnant adolescents to decrease interpersonal conflict, improve social support, and reduce perceived stress and depression offer the potential to serve as a novel approach to mitigate excess GWG. Consistent with this possibility, one cluster-randomized trial compared group prenatal care with usual individual care for improving reproductive outcomes among pregnant adolescents and young women (ages 14–21 years). In secondary analyses, the investigators found that those in group care showed greater reductions in perinatal depression symptoms [53] and had less GWG and less weight retention at 12-months postpartum [54]. In a separate study, a retrospective chart review analysis showed that pregnant adolescents who had received group prenatal care were more likely to have met GWG guidelines than adolescents who had received individual care [55]. While group prenatal care offers a promising approach to excess GWG in adolescent pregnancy, there are challenges to centering group care in pregnant teenagers. Group care can be difficult to coordinate in terms of timing of patients’ gestational age, appointment cancellations, and less overall flexibility to accommodate individual schedules.

Alternatively, interpersonal interventions to target social-emotional difficulties merit testing to address the problem of excess GWG in adolescent pregnancy by improving social functioning, perceived stress, and depression. Previous studies with adult pregnant women have shown that interpersonal interventions are associated with improvements in depression symptoms. For example, adult pregnant women assigned to a 16-week group-based interpersonal psychotherapy (IPT) program demonstrated greater improvements in depression symptoms as compared to those assigned to a parent education program [56, 57]. Moreover, adult pregnant women assigned to a culturally relevant, enhanced brief IPT (i.e., 8 individual sessions during pregnancy and maintenance sessions up to 6-months postpartum) showed improvements in social functioning and depression symptoms at 6-months postpartum, as compared to enhanced usual care [58].

### Study objectives

The current study is a randomized controlled pilot and feasibility study. The primary aim is to determine the feasibility and acceptability of a 6-session adolescent IPT program for prevention of excess GWG. We selected IPT based on its demonstrated effectiveness in reducing depression symptoms and improving social functioning in non-pregnant adolescents, including adolescents from diverse racial/ethnic groups and socioeconomic backgrounds, as well as pregnant adults [56–61]. Moreover, IPT has demonstrated effectiveness in non-pregnant adolescents in targeting other posited mechanisms for excess weight gain, such as emotional eating and binge-eating [62]. IPT is delivered as a relatively brief, manualized program using individual sessions every 2–3 weeks, throughout 12- to 30-weeks gestation. We are assessing feasibility and acceptability of IPT as delivered within the setting of an interdisciplinary adolescent pregnancy hospital clinic. The secondary aim is to estimate the effect of IPT integrated into usual prenatal care, in comparison to usual care only, on key healthcare outcomes grounded in the theoretical framework, including perinatal social functioning, perceived stress, depression symptoms, eating behavior, physical activity, sleep, GWG, delivery outcomes, and at 3-months postpartum, maternal weight retention, maternal/infant adiposity, and maternal insulin sensitivity.

## Methods/design

### Setting, inclusion criteria, and recruitment

Enrollment for the current study takes place at the Colorado Adolescent Maternity Program (CAMP) clinic at Children’s Hospital Colorado, University of Colorado School of Medicine/Anschutz Medical Campus in Aurora, Colorado. The CAMP clinic is an interdisciplinary adolescent perinatal program that includes an obstetrician/gynecologist, a nurse midwife, nurse practitioners, dieticians, social workers, psychologists, and case coordinators.

Inclusion criteria for the study are: [1] age 13–19 years [2]; pregnant, 12–18 weeks gestation; and [3] patient receiving care at the CAMP clinic. Exclusion criteria are [1] current full-syndrome psychiatric disorder that, in the opinion of the study investigators, would impede study compliance and/or necessitate more intensive treatment, such as conduct disorder, schizophrenia, or major depressive disorder with active suicidal ideation [2]; regular medication use likely to affect mood or weight, such as anti-depressants or stimulants [3]; high-risk pregnancy complications, such as preeclampsia, gestational diabetes, hypertension, multiple gestation, placenta previa, membrane rupture, or incompetent cervix [4]; major renal, hepatic, or endocrinological disorder, such as hyperthyroidism or Cushing syndrome, or a pulmonary disorder other than mild asthma; and [5] pre-pregnancy BMI < 5th percentile for age/sex.

The main recruitment method involves presenting the study in-person during prenatal intake visits to adolescents who appear to be eligible based upon review of the hospital electronic medical record. Trained research staff review the medical record as an initial screen of inclusion/exclusion criteria, including a review of adolescent age, gestational age, known pregnancy complications, medication use, and any major medical disorders that would be exclusionary. Adolescents who appear to be eligible based upon the medical record review are visited at an initial prenatal appointment by trained research staff, including nurses from the Perinatal Clinical and Translational Research Center (CTRC) at Children’s Hospital Colorado, who briefly describe the study, assess interest, and give patients a flyer and consent form to review. Participants who are interested after this initial visit are contacted by a study team member to schedule an initial screening visit at the clinic. Other recruitment methods include posting flyers in the clinic and mailing letters and flyers to CAMP patients.

### Consent

Research staff trained to work in research involving human subjects obtain written and active informed consent from potential research participants in a quiet, private room in the CAMP clinic at the screening/baseline study visit. Pregnant minors are able to consent themselves because, according to Colorado State Law, a pregnant minor is emancipated to approve prenatal, delivery, and post-delivery medical care for herself related to the intended live birth of a child (Colorado Revised Statute 13-22-103.5). Patients seeking services in the CAMP clinic typically attend appointments without a parent or guardian present; thus, requiring parental consent for participation would potentially exclude participants and bias findings. Additionally, study participation involves minimal risk. To participate in the 3-months postpartum maternal adiposity/insulin sensitivity assessments, participants must either be ≥ 18 years to provide informed consent or have a parent/guardian provide consent.

### Randomization and interventions

Adolescents who are determined to be eligible upon completion of the baseline assessment are randomized to IPT + usual CAMP clinic care or to usual care only. Randomization is stratified by age (13–16 years versus 17–19 years), weight status (normal weight, BMI 5–84th percentile versus overweight/obesity, BMI ≥ 85th percentile), race (Non-Hispanic White versus Other Race/Ethnicity), and baseline depression symptom level (Center for Epidemiological Studies-Depression Scale (CES-D) survey total score < 21 versus CES-D total score ≥ 21). Randomization strings were generated by an electronic program with permuted blocks.

#### CAMP usual care

Participants in both conditions continue to receive adolescent-focused interdisciplinary prenatal care at the CAMP clinic. Adolescent-focused prenatal care generally differs from adult-focused prenatal care by providing developmentally appropriate services that are tailored to meet the unique social-emotional needs of adolescents [63]. More specifically, adolescent-focused prenatal care addresses co-occurring psychosocial stressors that are more prevalent among pregnant adolescents as compared to pregnant adults, such as mental health problems, substance abuse and use, school problems, family conflict, and poverty [35]. At the CAMP clinic, in addition to usual obstetrical prenatal care, CAMP patients are seen a minimum of 4 times by clinic social workers, once for a psychosocial intake and 3 additional times during pregnancy. Additional social worker sessions are scheduled as needed. These meetings provide information about mood changes in pregnancy and basic education on postpartum depression. All patients also meet once with a dietician during the first trimester to review healthy weight gain and nutrition in pregnancy. Additional dietician appointments are scheduled if concerns about inadequate weight gain emerge or in the event of significant nausea and/or vomiting.

#### Interpersonal psychotherapy (IPT)

Adolescents randomized to IPT + usual care also are scheduled for the IPT program, “Healthy Relationships, Healthy Weight.” This program was adapted from the IPT for the Prevention of Excess Weight Gain (IPT-WG) manual, which was designed for use in non-pregnant adolescents at risk for excess weight gain because of above-average weight and disordered eating/loss-of-control eating patterns [62]. The IPT-WG program is a 12-session group intervention based on the interpersonal theoretical model, which posits that conflictual interactions and lack of social support can worsen or maintain negative affect [64]. Negative affect, in turn, can lead to overeating when not hungry or overeating highly palatable, energy-dense foods (e.g., sweets and snacks), which taken together, lead to excess weight gain [64].

The adaptation of IPT-WG for the current study includes 3 key modifications. First, sessions are delivered in an individual versus weekly group format to facilitate flexible scheduling. Facilitators make every effort to schedule in-person sessions so that they occur immediately before or after usual prenatal care appointments at the CAMP clinic to minimize participant burden. Depending on participant preference, facilitators are available to schedule sessions at the CAMP clinic during normal business hours, independent of usual prenatal care. Moreover, sessions are scheduled once every 2–3 weeks starting after the baseline and screening visit and ending by 28–30 weeks gestation, thus providing support to adolescents throughout their pregnancy. We chose not to offer telephone sessions in this pilot feasibility study in order to explicitly evaluate feasibility and acceptability of delivering an in-person program within an interdisciplinary adolescent perinatal healthcare setting. Second, the number and duration of sessions is decreased from 12 90-minute sessions to 6 60-minute sessions. This change was based upon preliminary survey data from CAMP clinic patients that indicated a preference for 6 or fewer sessions. Finally, session content is specifically tailored to address typical issues associated with adolescent pregnancy.

A summary of session content for this adaptation of IPT for the prevention of excess GWG is provided in Table 1. The first session focuses on psychoeducation about the connections among social relationships, mood, eating behavior, and healthy weight gain in pregnancy. This initial session also includes a brief interpersonal inventory to assess adolescents’ current relationships and to formulate explicit program goals centered on addressing conflict and support in current relationships. The second session focuses on communication analysis, especially nonverbal aspects of communication. The third, fourth, and fifth sessions involve learning new communication skills and applying these communication skills to current relationships with an aim to increase support or reduce conflict. Finally, the sixth session involves planning for using new communication skills in the context of the transition of delivery and psychoeducation about help-seeking in the future. Participants are encouraged to apply skills learned in each session to current relationships in between sessions through home practice exercises. These home practice exercises are facilitated by a binder with handouts. Facilitators call and/or text message participants in between sessions to check-in and remind them to complete home practice and attend the following session.

**Table 1:** Summary of IPT session content for the randomized controlled pilot study protocol

Session	Content
1	Introduction; psychoeducation about weight gain in pregnancy; theoretical model of social relationships, mood, and eating patterns; interpersonal inventory; identify program goals; assign daily journal
2	Affective expression, communication analysis
3	New communication skills: “Strike while the iron is cold,” “Using ‘I’ statements,” and “Be specific.” Script and role-play a conversation with new skills to be assigned as home practice before next session
4	New communication skills: “Put yourself in their shoes” and “What you don’t say speaks volumes.” Script and role-play a conversation with skills to be assigned as home practice before next session
5	New communication skills: “Have a few solutions in mind” and “Don’t give up.” Script and role-play a conversation with skills to be assigned as home practice before next session
6	Program review; planning ahead for transition of delivery and caring for baby; graduation

IPT sessions are facilitated by a clinical psychologist or by a graduate student in clinical/counseling psychology, marriage and family therapy, or related field. All intervention facilitators are trained by a program developer (LBS) in the administration of the IPT program and receive clinical supervision on audio-recorded sessions from a program developer and licensed clinical psychologist.

At the beginning of each session, participants’ height and weight are measured and participants also report their mood on a Mood Monitoring Questionnaire. Any participant whose mood worsens considerably or who develops a psychiatric issue necessitating additional treatment is immediately referred. In the event of an acute psychiatric crisis (e.g., active suicidal ideation), the on-call child psychiatrist at Children’s Hospital Colorado is paged for an immediate consult, evaluation, and possible referral to the Emergency Department.

### Outcomes

Primary and secondary outcomes are summarized in Table 2.

**Table 2:** Overview of constructs and assessments throughout the randomized controlled pilot study protocol

Construct	Measurement	Description	Intervals assessed	Reference
Primary outcomes				
Feasibility of study		Number of eligible participants, randomized participants, retention and attrition of randomized participants	Baseline, mid-pregnancy, post-program, 3-month postpartum	[65]
Acceptability of IPT		IPT session attendance (≥ 80%), above-average participant IPT program ratings	Post-program	[66]
Secondary Outcomes				
Social functioning	SAS-SR	24-item self-report scale of interpersonal functioning in family, friend, romantic, and school or work domains	Baseline, mid-pregnancy, post-program, 3-month postpartum	[67]
	NRI-BSV	28-item self-report scale of relationship characteristics for mother, father, peer, and romantic partner relationships		[68]
Perceived stress	PSS	14-item self-report scale to assess perception of stress	Baseline, mid-pregnancy, post-program, 3-month postpartum	[69]
Depression	CES-D	20-item self-report scale to assess depression symptoms	Baseline, mid-pregnancy, post-program, 3-month postpartum	[70]
	EPDS	10-item self-report scale to assess depression symptoms	Baseline, mid-pregnancy, post-program, 3-month postpartum	[71, 72]
	MINI-KID	Structured clinical interview to assess psychiatric disorders, such as major depressive disorder	Baseline, post-program, 3-month postpartum	[73]
Disinhibited eating	EDE	Semi-structured interview to assess disordered eating including objective binge, subjective binge, and objective overeating	Baseline, post-program, 3-month postpartum	[74, 75]
	EES-C	25-item self-report questionnaire to assess eating in response to negative emotions	Baseline, mid-pregnancy, post-program, 3-month postpartum	[76]
Physical activity	ActiGraph GT3X+	Body-worn accelerometer to measure 7 days and nights of habitual physical activity including step counts, light and moderate-vigorous intensity, and sedentary time	Baseline, mid-pregnancy, post-program	[77, 78]
Sleep disturbance	ActiGraph GT3X+	Body-worn accelerometer to measure 7 nights of sleep including total sleep time, sleep onset latency, wake after sleep onset, and sleep efficiency	Baseline, mid-pregnancy, post-program	[79, 80]
Body composition	BMI indices	Height and weight measured to calculate BMI	Baseline, mid-pregnancy, post-program, 3-month postpartum	[81]
Maternal postpartum adiposity	Body fat	DXA conducted to measure body composition including total fat and lean mass	3-month postpartum	[82, 83]
Maternal insulin sensitivity	WBISI, QUICKI, HOMAIR	7-sample, 2-h oral glucose tolerance test to estimate insulin sensitivity	3-month postpartum	[84, 85]
Infant adiposity	Body fat	Infant PeaPod conducted to measure body composition including total fat and lean mass	3-month postpartum	[86]

*Baseline* baseline/screening assessment occurring around 12–18 weeks gestation; *Mid-Pregnancy* mid-pregnancy assessment occurring around 21–28 weeks gestation; *Post-Program* post-program assessment occurring around 30–34 weeks gestation; *3 Month Postpartum* assessment occurring around postpartum week 12; *SAS-SR* Social Adjustment Scale, Self-Report; *NRI-BSV* Network of Relationships Inventory-Behavioral Systems Version; *PSS* Perceived Stress Scale; *CES-D* Center for Epidemiologic Studies-Depression Scale; *EPDS* Edinburgh Postnatal Depression Scale; *MINI-KID* Mini-International Neuropsychiatric Interview for Children and Adolescents; *EDE* Eating Disorder Examination; *EES-C* Emotional Eating Scale-Adapted for Children; *BMI* body mass index (kg/m^2^, *z*-score, percentile); *DXA* dual-energy X-ray absorptiometry; *WBISI* whole body insulin sensitivity index; *QUICKI* quantitative insulin sensitivity check index; *HOMAIR* homeostasis model assessment of insulin resistance

#### Primary outcomes

##### Feasibility of study

Feasibility will be defined according to several metrics, including feasible recruitment, enrollment, and retention. Estimates are derived from previous longitudinal studies with pregnant women [65, 87–90]. Feasible recruitment will be defined as identifying at least 480 potentially eligible patients based upon medical record review by research staff to approach during the prenatal intake visit. Feasible enrollment will be defined as approximately 25% (120/480) of preliminary eligible patients agreeing to schedule a screening/baseline assessment and about 67% (80/120) attending the assessment and enrolling in the study. Feasible enrollment will be estimated as 60% (50/80) of enrolled patients being eligible to be randomized. Finally, feasible retention will be assessed as at least 75% (38/50) of randomized participants successfully completing at least three of four assessment intervals, including the baseline/screening assessment. This definition of feasible retention refers to the assessment intervals only and does not include the IPT sessions.

##### Acceptability of IPT

Acceptability will first be measured by IPT session attendance. Acceptable IPT session attendance will be defined by 50% of participants randomized to IPT attending 5 or 6 (≥ 80%) of the total 6 sessions [87–90]. Acceptability will also be measured by participant ratings on a program acceptability interview adapted for the current study from the Treatment Process Questionnaire and administered by a project staff at the end of treatment assessment [66]. This questionnaire asks participants to report on their reasons for enrolling in the study, what they liked and did not like about the IPT program and the perceived impact of the IPT program on their mood and health. Acceptable participant ratings will be defined as above-average ratings.

#### Secondary outcomes

##### Social functioning, perceived stress, and depression

Participants complete self-report questionnaires assessing social functioning, perceived stress, and depression on a computer through Research Electronic Data Capture (REDCap). A project staff member reviews completed questionnaires for missing items before the conclusion of the study visit and invites participants to respond to those missing items.

Social functioning is measured by two questionnaires. The Social Adjustment Scale-Self-Report (SAS-SR) is a 23-item questionnaire assessing social role functioning in several domains including peer, family, romantic relationships, and school/work [67]. Higher scores indicate worse social functioning. The Network of Relationships Inventory-Behavioral Systems Version (NRI-BSV) is a 28-item questionnaire assessing five social support features and three negative interaction features [68]. Stress is assessed as a continuous measure of stress perception by the Perceived Stress Scale (PSS), a 10-item questionnaire [69]. Higher scores suggest greater stress perception. Depression is measured by two questionnaires. The Center for Epidemiological Studies-Depression Scale (CES-D), a 20-item questionnaire assessing depression as a continuous measure of symptoms [70]. Depression is also assessed as a continuous measure of symptoms on the Edinburgh Postnatal Depression Scale (EPDS), a 10-item questionnaire [71, 72]. Higher scores on the CES-D and the EPDS are indicative of more elevated depression symptoms. Finally, depression diagnosis is assessed via the Mini-International Neuropsychiatric Interview for Children and Adolescents (MINI-KID), a structured diagnostic interview for psychiatric disorders in children and adolescents.

##### Eating behavior

The overeating section of the Eating Disorders Examination (EDE) Version 14.0, a semi-structured clinical interview [74, 75], is administered by trained research staff to assess overeating episodes with and without a feeling of subjective loss-of-control. The EDE has demonstrated good reliability and validity in adolescent samples [91]. Emotional eating, referring to eating in response to negative emotions, is assessed with the Emotional Eating Scale-Adapted for Children and Adolescents (EES-C) [76]. The EES-C is a 25-item self-report questionnaire that has shown good psychometric properties in adolescent samples [76].

##### Physical activity and sleep disturbance

Habitual physical activity, sedentary behavior, and sleep disturbance are derived from ActiGraph GT3X+ accelerometers (ActiGraph, Pensacola, FL), which have well-established validity and reliability in adolescents [77, 79]. These lightweight monitors are worn for seven days and nights on participants’ non-dominant wrist and are only removed for water-based activities, such as showering or swimming. Accelerometers record data with a sampling frequency of 30 Hz using 60-second epochs. Light physical activity, moderate-to-vigorous activity, and sedentary time are classified based on mean counts per epoch according to established guidelines [78]. Parameters of sleep disturbance include total sleep time, sleep onset latency, wake after sleep onset, and sleep efficiency [80].

##### Gestational weight gain

Weight is derived to the nearest 0.1 kg on a calibrated digital scale, and height is derived by a stadiometer in triplicate. Weight and height will also be derived from the medical record for intervals that do not coincide with a study assessment interval. More specifically, weight and height will be derived from the medical records at prenatal appointments, at delivery, and at postnatal appointments. Continuous GWG during pregnancy will be measured using two metrics: [1] GWG during the experimental phase, as defined by the difference between measured maternal weight at the baseline assessment to the post-program assessment, and [2] total GWG, as defined by the difference between self-reported pre-pregnancy weight and maternal weight at delivery, derived from the medical record. Classification of pre-pregnancy weight status as normal weight, overweight, or obese will be based on CDC BMI percentile for age [81]. Categorical GWG, defined as appropriate versus excessive, will be measured relative to Institute of Medicine guidelines for gestational age for both GWG during the experimental phase and total GWG.

##### Maternal postpartum weight retention

Continuous maternal postpartum weight retention will be measured as the difference in self-reported pre-pregnancy weight and measured maternal weight at the 3-month postpartum assessment [39].

##### Maternal postpartum adiposity

For those who participate in the adjunct metabolic assessments, participants have a dual energy X-ray absorptiometry (DXA) scan to evaluate body composition, including total lean mass and total fat mass, using Hologic QDR Discovery A (S/N81337; Bedford, MA) [82]. DXA has demonstrated good validity in youth [83].

##### Maternal postpartum insulin sensitivity

Maternal insulin sensitivity is derived from a 2-hour oral glucose tolerance test at the 3-month postpartum interval. In the morning following a 10-hour overnight fast, participants ingest 1.75 g/kg of glucola (max = 75 g). Blood is sampled via an intravenous line for insulin, glucose, and c-peptide at fasting, 10, 20, 30, 60, 90, and 120 min after ingesting the glucola [85]. Insulin sensitivity will be estimated with the whole body insulin sensitivity index (WBISI), as well as the quantitative insulin sensitivity check index (QUICKI) and the homeostasis model assessment of insulin resistance (HOMA-IR). These methods of measuring insulin sensitivity have been validated against euglycemic-hyperinsulinemic clamp-derived measures in youth with overweight and obesity [84].

##### Infant adiposity

At the 3-month postpartum assessment, infants will complete a PEAPOD to evaluate fat mass and fat-free mass using air displacement plethysmography [86].

### Participant timeline

All assessments take place at the CAMP clinic, with the exception of the 3-month postpartum assessment, which takes place at the Outpatient Pediatric CTRC at Children’s Hospital Colorado. Participant timeline is presented in Table 3.

**Table 3:** Overview of a participant’s timeline, assessment intervals, and core measures

	Study Period											
	Initiation of prenatal care	Allocation	Intervention						Post-intervention			3-month post-partum
**Gestational week**		12–18	24	26	28	30	32	34	36	38	40	
Enrollment												
Eligibility Screen	X											
Informed Consent		X										
Allocation		X										
Interventions												
IPT			X	X	X	X	X	X				
UC												
Medical	X	X	X	X	X	X	X	X	X	X	X	X
Nutrition	X			X								
Social work	X			X					X			
Assessments												
Feasibility	X	X	X	X	X	X	X	X	X			X
Acceptability	X	X	X	X	X	X	X	X	X			
Social functioning		X			X				X			X
Perceived stress		X			X				X			X
Depression	X	X	X	X	X	X	X	X	X			X
Eating behavior		X			X				X			X
Physical activity		X			X				X			
Sleep disturbance		X			X				X			
Height/weight	X	X	X	X	X	X	X	X	X	X		X
Maternal insulin sensitivity												X
Maternal adiposity												X
Infant adiposity												X

*IPT* interpersonal psychotherapy, *UC* usual care

#### Screening/baseline assessment

Potentially eligible and interested volunteers are invited to participate in a screening and baseline assessment appointment. This appointment takes approximately three hours. A trained research staff member obtains informed written consent. Participants complete online questionnaires via REDCap to assess social functioning, perceived stress, depression symptoms, and eating behaviors. A project staff member administers a structured clinical interview to rule out the presence of a psychiatric disorder, current self-injurious behavior, or current suicidal behavior that would warrant more intensive treatment. Participants who meet criteria for a current full-syndrome psychiatric disorder or who report current and active suicidal ideation are immediately referred to CAMP behavioral health staff for further evaluation and treatment. At the screening visit, project staff also administer a semi-structured interview to assess eating patterns, including overeating and binge eating, and review a 3-day diet record. Research staff who administer clinical interviews are trained by clinical psychologists through a combination of didactics, role-playing, and live supervision. Clinical interviews are audio-recorded and reviewed by clinical psychologists on the research team for fidelity to the interview protocol. At the end of the screening visit, participants are fitted with an ambulatory accelerometer that they are asked to wear for seven days following the study visit to measure habitual physical activity. Participants are paid $50 for completing this screening visit. At the end of the screening visit, eligible participants are randomized to either IPT + usual care or usual care only.

#### Mid-program assessment

Participants complete a brief assessment, estimated to take no longer than 30 min, halfway through the experimental phase, corresponding to about 6–9 weeks after the baseline assessment. Participants repeat questionnaires assessing social functioning, stress, depression symptoms, and eating behaviors. They also re-wear the accelerometer for seven days following the study visit to re-assess habitual physical activity. Participants receive a pack of newborn diapers for completing this mid-program assessment.

#### Post-program assessment

Participants complete an assessment at the end of the experimental phase, which corresponds to about 12–18 weeks after the baseline assessment and just prior to delivery. This assessment takes about two hours. Participants repeat the same research tasks as at the baseline assessment. In addition, a project staff member administers a program acceptability interview to adolescents who were randomized to the IPT condition. Participants are paid $75 for completing the post-program assessment.

#### 3-month postpartum assessment

Participants complete a final assessment at 3-months postpartum, a time when inter-individual differences in postpartum weight retention and postpartum depression emerge [92]. Participants repeat the same research tasks at the baseline assessment. Participants’ 3-month old babies complete an infant body composition assessment using air displacement plethysmography (PEAPOD). A project staff member reviews the medical record to assess measured height and weight at delivery and postpartum appointments, as well as any pregnancy or birth complications. Participants are paid $75 for completing the 3-month postpartum assessment.

Participants are invited to opt in, or out, of an additional adjunct metabolic study, which includes a maternal DXA scan of post-partum adiposity and a 2-h oral glucose tolerance test (OGTT) to estimate maternal post-partum insulin sensitivity. Participants are paid $75 for the adjunct metabolic procedures.

### Sample size

We plan to randomize 50 out of 80 (60%) enrolled participants following a screening/baseline eligibility assessment. Our estimation of a total sample size of *N* = 50 (i.e., *n* = 25 per condition) is based on recommendations for a two-arm superiority pilot trail in which small-to-moderate standardized effects are anticipated [93]. Furthermore, our sample size estimation is consistent with recommendations for pilot randomized trials using an 80% one-sided confidence interval approach to exclude a clinically important difference between study arms [94].

### Statistical analysis

Baseline participant characteristics and feasibility/acceptability findings will primarily be measured with descriptive statistics, including mean with standard deviation, median with interquartile range, frequency, and percentage. Analysis of covariance (ANCOVA) will be used to evaluate group differences (IPT + usual care versus usual care) in secondary outcomes, including social functioning, perceived stress, depression symptoms, eating behaviors, physical activity, sleep disturbance, maternal post-partum insulin sensitivity, maternal post-partum adiposity, and infant adiposity. We will use 95% confidence intervals to measure precision of the estimated differences between conditions, per recommendations [95]. Given that this is a pilot study, results will be considered preliminary. Although our theoretical framework suggests that IPT + usual care, as compared to usual care only, will impact these secondary outcomes via a series of mechanistic pathways, this pilot feasibility study is not sufficiently powered to test mediation. Covariates considered in these models will include baseline maternal age, gestational age, race/ethnicity, baseline CES-D score, and pre-pregnancy BMI/weight status. Missing data will be examined for patterns of missingness and imputation, with the intent-to-treat sample, will be used if deemed appropriate. Given the pilot nature of the study, we also will conduct sensitivity analyses using listwise deletion with complete data.

### Ethics and dissemination

The protocol has been approved by a single Institutional Review Board, the Colorado Multiple Institutional Review Board (COMIRB), which provides oversight for the University of Colorado School Of Medicine and Children’s Hospital Colorado. An Institutional Review Board authorization agreement was established between the Institutional Review Board of Colorado State University, an administrative site, and COMIRB, in order for COMIRB to serve as the primary IRB. We follow Institutional Review Board requirements pertaining to reporting of unanticipated problems and adverse events.

Personal information is collected directly from research participants and from their medical record. Personal information is stored separately from other research data and will not be shared with anyone outside of the research team, which includes personnel at the University of Colorado, Children’s Hospital Colorado, and Colorado State University, with the exception of entities that monitor human subject research, such as COMIRB. Electronic data are stored and managed by REDCap and a secure server at the University of Colorado. The REDCap system is password protected and only accessible by research staff members. Digital audio recordings from interviews and interventions are uploaded to password-protected folders on a secure server at the University of Colorado. After uploading, recordings are deleted from the digital recording device. Paper data are stored in locked cabinets in administrative research space at Children’s Hospital Colorado. All data will be preserved for seven years following IRB acknowledgement of study closure. Results and conclusions from the current study will be disseminated by publication in peer-reviewed journals and conference presentations. We also will disseminate findings to clinic providers who have been involved in supporting the study.

## Discussion

The current study is a randomized controlled feasibility study piloting a novel approach to the prevention of excess GWG in adolescent pregnancy. The primary aim is to assess the feasibility and acceptability of a relatively brief, 6-session individual IPT program, delivered within the context of an interdisciplinary adolescent pregnancy hospital clinic. The secondary aim is to explore whether there are health benefits to pregnant adolescents who receive IPT—in perinatal social functioning, perceived stress, depression symptoms, eating behaviors, physical activity, sleep, GWG, and postpartum maternal and infant metabolic health—as compared to the usual care that pregnant adolescents would receive in an interdisciplinary adolescent maternity clinic.

Preventative interventions for excess GWG in adult women have previously focused on lifestyle modification, including healthy eating and physical activity. The available literature suggests that lifestyle-based interventions require significant financial and time resources, and unfortunately, generally demonstrate insufficient effectiveness [29]. Moreover, no study to date has been specifically designed to evaluate a prevention of GWG intervention in pregnant adolescents, even though excess GWG is prevalent in this population and is associated with serious, negative health outcomes [3]. Pregnant adolescents face unique socioemotional needs, often including mental health problems, academic problems, family conflict, and poverty [35]. There is growing evidence that such social-emotional factors may interfere with a healthy weight gain in pregnancy through their influence on overeating to cope with negative emotions and stress, reduced physical activity, and disturbed sleep [96, 97]. From an interpersonal theoretical framework, receipt of an IPT intervention is posited to reduce conflict and increase support in adolescents’ interpersonal relationships, which in turn, is theorized to improve perceived stress and negative affect, and ultimately facilitate healthy eating, physical activity, and sleep to influence a healthy GWG.

The proposed pilot randomized feasibility study is the first necessary step in understanding the potential utility of an evidence-based psychotherapy intervention—addressing social functioning, stress, and negative emotions—for prevention of excess GWG in adolescent pregnancy. We anticipate that IPT will be feasible and acceptable to pregnant adolescents. Moreover, we have preliminary data that suggest it will be feasible to incorporate a psychosocial intervention into an interdisciplinary hospital clinic, as evidenced by a 72% attendance rate in an existing parenting skills training intervention in the CAMP clinic. However, it is possible that there could be obstacles to attendance (e.g., transportation). Some we have anticipated (e.g., providing taxis and bus passes as needed), but others (e.g., school/work schedules) could interfere with sessions. A strength of the individualized nature of our IPT program is that it permits a high degree of flexibility in rescheduling sessions at alternative times.

With respect to secondary outcomes, we anticipate that adolescents who receive IPT, relative to usual care only, will show patterns of improved perinatal social functioning, lower perceived stress, reduced depression symptoms, healthier eating patterns, increased physical activity, improved sleep, less excess GWG, and better postpartum maternal and infant metabolic health. However, IPT may improve social-emotional adjustment without preventing excess GWG or postpartum weight retention and metabolic outcomes. In this case, we would have initial evidence for a brief, potentially cost-effective approach to improving social-emotional health in pregnant adolescents, which has important public health relevance for postpartum depression prevention and maternal-infant health, given the serious, negative effects of depression on maternal-infant psychosocial outcomes [98]. Ultimately, results and conclusions have the potential to benefit not only emotional and physical health outcomes for adolescent mothers, but also health outcomes for offspring as part of a larger public health perspective on the intergenerational transmission of depression, obesity, and cardiometabolic disease.

Feasibility and acceptability findings from the current pilot study will inform the development of a larger randomized controlled trial that is adequately powered to measure the effectiveness of this brief individual psychosocial intervention to prevent excess GWG in adolescents. Testing the effectiveness of this psychosocial intervention within the structure of an existing interdisciplinary adolescent maternity hospital clinic is ideal to facilitate successful transfer of research-based knowledge to routine clinical practice. The potential for dissemination of this psychosocial intervention is further enhanced by evidence showing that IPT can be delivered effectively and with fidelity by trained facilitators who do not necessarily have an advanced degree in psychology, such as lay community health workers [99].

We considered the selection of the control group extensively. A limitation of our usual care control is that we will not be able to account for potential confounding effects of attention. Because adolescent patients receive education about depression as part of prenatal usual care, and because we seek to first establish feasibility and acceptability, we felt that comparison to usual care was the optimal, initial step. Depending upon the results, a subsequent well-powered trial might include a 3-group design comparing [1] IPT to [2] usual care and either (3a) an attention-matched educational control or (3b) a standard behavioral lifestyle intervention. Due to the pilot nature of this study, one shortcoming is that we will only have maternal insulin sensitivity and maternal/infant adiposity at postpartum, and thus, will not be able to control for earlier levels of these variables. These measures are secondary outcomes, and if positive signals are detected from these pilot data, future studies might consider use of repeated measures of maternal metabolic characteristics and measures at birth of infant adiposity. Another limitation of this pilot study is that outcome assessors are not blind to intervention assignment, and thus, assessment of some outcomes may be influenced by expectancy biases (i.e., such as those measured by clinical interview). That being said, a potential strength of the current study is heterogeneity of outcome variables, either by participant self-report or objective measures, such as accelerometers to assess habitual physical activity and sleep. A subsequent randomized controlled effectiveness trial would have the resources to ensure that research study staff who collect outcome data are blind to intervention assignment.

The concept of focusing on pregnant adolescents will allow us to target an underrepresented, underserved group who are likely to face heightened social-emotional challenges as compared to pregnant adult women [35]. Opportunities to intervene with this at-risk group can be expected to help prevent many of these young women, and their future infants, from a lifetime trajectory of obesity with its multiple adverse health consequences. The proposed IPT intervention represents a novel approach to addressing excess GWG in adolescents by targeting psychosocial risk factors including social functioning, perceived stress, and depression symptoms.

## Data Availability

The de-identified dataset generated from this protocol will be available from the corresponding author upon reasonable request.

## Abbreviations

ANCOVA: Analysis of covariance
CAMP: Colorado Adolescent Maternity Program
CES-D: Center for Epidemiological Studies-Depression Scale
COMIRB: Colorado Multiple Institutional Review Board
CTRC: Clinical and Translational Research Center
DXA: Dual energy X-ray absorptiometry
EDE: Eating disorders examination
EES-C: Emotional Eating Scale for Children
EPDS: Edinburgh Postnatal Depression Scale
GWG: Gestational weight gain
HOMA-IR: Homeostasis model assessment of insulin resistance
IPT: Interpersonal psychotherapy
MINI-KID: Mini-International Neuropsychiatric Interview for Children and Adolescents
NRI-BSV: Network of Relationships Inventory-Behavioral Systems Version
OGTT: Oral glucose tolerance test
PSS: Perceived Stress Scale
QUICKI: Quantitative insulin sensitivity check index
REDCap: Research Electronic Data Capture
SAS-SR: Social Adjustment Scale-Self Report
WBISI: Whole body insulin sensitivity index
